# Supplementary material for: The impact of civil unrest on child health care: Evidenced by acute medical complications at presentation – A retrospective comparative study
Source: PLoS One. 2025 Apr 9;20(4):e0320902. doi: 10.1371/journal.pone.0320902 (PMC11981157; doi:10.1371/journal.pone.0320902)
Supplement: S1 Table — (DOCX) [file pone.0320902.s002.docx]

| **S_1 Table. Types of acute Medical complications at presentation. Bahir Dar University Tibebe-Ghion Hospital.** | | | | |
| --- | --- | --- | --- | --- |
| **S.No** | **Type of acute Medical complication** | **Prior to unrest** | **Into the unrest** | **Total** |
|  | **No acute Medical complication at presentation** | **269** | **159** | **428** |
|  | Pyogenic meningitis | 0 | 3 | 3 |
|  | Increased ICP | 1 | 3 | 4 |
|  | Brain abscess | 1 | 1 | 2 |
|  | Ventriculitis | 1 | 2 | 3 |
|  | Acute motor deficit | 3 | 3 | 6 |
|  | Status epilepticus | 1 | 3 | 4 |
|  | SIADH Secretion | 0 | 1 | 1 |
|  | Subdural collection | 0 | 2 | 2 |
|  | Hydrocephalus | 2 | 2 | 4 |
|  | Quadriplegia + Status epilepticus | 1 | 0 | 1 |
|  | Increased ICP + Status epilepticus | 0 | 1 | 1 |
|  | Anemia + status epilepticus | 0 | 1 | 1 |
|  | Hemiplegia + seizure + Brain abscess | 0 | 1 | 1 |
|  | Ruptured MMC | 1 | 1 | 2 |
|  | Infected MMC | 1 | 0 | 1 |
|  | Acute motor deficit + ICH | 0 | 1 | 1 |
|  | **Neurologic Complications** | **12** | **25** | **37** |
|  | Para pneumonic collection | 6 | 8 | 14 |
|  | Para pneumonic and pericardial collection | 0 | 2 | 2 |
|  | Air leak syndrome | 0 | 2 | 2 |
|  | Pneumonia | 0 | 2 | 2 |
|  | Upper airway obstruction/ Bacterial thrachitis | 0 | 1 | 1 |
|  | Respiratory failure | 0 | 1 | 1 |
|  | **Respiratory Complications** | **6** | **16** | **22** |
|  | Congestive Heart Failure | 12 | 24 | 36 |
|  | CHF with Respiratory failure | 0 | 1 | 1 |
|  | CHF with Pulmonary edema | 0 | 1 | 1 |
|  | CHF with Cardiogenic shock | 0 | 1 | 1 |
|  | CHF with Pulmonary edema and AKI | 0 | 1 | 1 |
|  | Infective endocarditis | 1 | 4 | 5 |
|  | Galloping infection (lung and pericardium) | 0 | 1 | 1 |
|  | **Cardiac Complications** | **13** | **33** | **46** |
|  | Perforated appendicitis/abscess | 1 | 14 | 15 |
|  | Gangrenous intussusception | 0 | 4 | 4 |
|  | Gangrenous intussusception + shock | 0 | 1 | 1 |
|  | Peritonitis | 0 | 1 | 1 |
|  | Necrotic rectal prolapse | 0 | 1 | 1 |
|  | **GI Complications** | **1** | **21** | **22** |
|  | Compartment syndrome | 0 | 1 | 1 |
|  | Wet gangrene | 1 | 2 | 3 |
|  | Oral candidiasis | 0 | 1 | 1 |
|  | Pyomyositis | 0 | 1 | 1 |
|  | Infected burn | 0 | 1 | 1 |
|  | Sepsis with cellulitis | 0 | 1 | 1 |
|  | **Musculoskeletal complications:** | **1** | **7** | **8** |
|  | Severe anemia | 4 | 11 | 15 |
|  | Severe anemia + CHF | 0 | 1 | 1 |
|  | DVT/deep vein thrombosis | 0 | 2 | 2 |
|  | **Hematologic and vascular Complications** | **4** | **14** | **18** |
|  | Hydrocele abscess | 1 | 0 | 1 |
|  | Fournier’s gangrene | 0 | 1 | 1 |
|  | Incarcerated Right Inguinal hernia | 0 | 2 | 2 |
|  | Peri-nephric abscess | 0 | 1 | 1 |
|  | Post circumcision phimosis | 0 | 1 | 1 |
|  | Acute Kidney Injury/ AKI | 1 | 1 | 2 |
|  | AKI + Dehydration | 0 | 1 | 1 |
|  | **Genito-urinary and renal disorders** | **2** | **7** | **9** |
|  | Dehydration | 1 | 6 | 7 |
|  | Paralytic ileus | 1 | 3 | 4 |
|  | Sepsis | 1 | 5 | 6 |
|  | Septic shock | 1 | 4 | 5 |
|  | Electrolyte derangement | 0 | 2 | 2 |
|  | DHN with electrolyte abnormalities | 0 | 2 | 2 |
|  | DHN + GI Onset sepsis | 0 | 1 | 1 |
|  | **Fluid and electrolyte abnormalities** | **4** | **23** | **27** |
|  | Metabolic acidosis | 0 | 1 | 1 |
|  | Diabetic ketoacidosis /DKA | 4 | 10 | 14 |
|  | **Metabolic Disorders** | **4** | **11** | **15** |
|  | **Total** | **316** | **316** | **632** |
