# Supplementary material for: The impact of civil unrest on child health care: Evidenced by acute medical complications at presentation – A retrospective comparative study
Source: PLoS One. 2025 Apr 9;20(4):e0320902. doi: 10.1371/journal.pone.0320902 (PMC11981157; doi:10.1371/journal.pone.0320902)
Supplement: Attachment — Submitted filename: PONE_Review_ChildHealthcare_Conflict_Aug2024.docx [file pone.0320902.s003.docx]

**Review: The Impact of civil unrest on child health care: evidenced by acute medical complications at presentation – a retrospective comparative study.**

Overall:

- Do a careful reading for grammatical corrections

Abstract:

- .

Background/Introduction:

- You provide a well-written perspective on how conflict affects the physical infrastructure of health facilities, and transport systems. This description would benefit from information about what happens to healthcare providers during conflict in your country context – are they expected to continue working? Are they evacuated? Are they all moved to work at the tertiary facilities – do clinics close but hospitals stay open? I think adding this information specific to your own country context would be helpful. If there are no peer-reviewed publications on this topic you can use UN or government reports and cite those. You can even include your own experience – look online for how to add this option as a citation or ‘reference’ based on the required referencing style.
- Final sentence – most importantly is the need to inform the government about how to maintain healthcare provision in times of civil unrest.

Methods:

- Study design – you have included a lot of information in this section about the statistical analysis, which belongs in the statistical analysis section. For the study design section, it’s okay to be brief and define this as a retrospective medical record review.
- Study design – your ethics statement is in this section, but usually would have its own section or be shown separately in a section after the manuscript. Please review journal requirements and include as advised by the journal
- Definition – acute medical complications: There’s nothing in the definition to indicate this can be a result of delayed care, but I believe this is the premise of your paper; the care is delayed and as a result, children are more likely to have acute medical complications? If correct, please refine your definition to include a reference to delayed care.
- Sample size – I don’t believe you need the full equations included. You can make this section far more concise by using wording only. See other journal articles for how others have done this.
- Table 1 – please provide table footnotes on what Beta represents

Results:

- Table 2. Please remove color stripes from the table. Please add column percentages so that it’s easier to ‘see’ the data distribution between groups
- “table 1” in STATA (?) output – please remove this and create “Table 3”. In the narrative please remove definitions and just describe the findings objectively.
- “table 2” in STATA (?) output – please remove this and create “Table 4” – if possible, consider merging these two STATA tables into a single table for the results. Think how best to share your findings in a way that’s both understandable and concise
- “table 3” and “table 4” in STATA output. Remove the tables and report in the narrative
- “Interpretation” – remove this statement and put everything into narrative that describes your findings.
- “table 5” in STATA output – remove this as well and describe in the narrative.

Discussion:

- You need to describe a bit more about how the other research studies have documented negative health effects if not at the individual level – what did they look at that was not as rigorous as your study?
- In the third paragraph which starts “the acute medical complications” – please describe a bit more about the differences between rural and urban before and during conflict – it doesn’t come out clearly here

Limitations:

- Remove the word ‘holistically’ – be more concise; you can just say that because the study was done at one tertiary facility, the results may not be generalizable to other conflict settings

Conclusion, Recommendation, Implications

- There are too many section here. You should have your “Discussion” and “Conclusion” sections. Your implications all need to be incorporated into your discussion section, connected to the key findings that you shared in the results section. Aim to have about 3-4 main points (1 per paragraph) and work the implications into each paragraph so you have a coherent discussion that flows well. Your limitations section should be the final paragraph in your discussion.
